# Are Psychosocial Resources Buffering the Relation Between Physical Work Behaviors and Need for Recovery?

**DOI:** 10.3389/ijph.2022.1604787

**Published:** 2022-12-16

**Authors:** Margo Ketels, Thomas Belligh, Dirk De Bacquer, Els Clays

**Affiliations:** ^1^ Department of Public Health and Primary Care, Faculty of Medicine and Health Sciences, Ghent University, Ghent, Belgium; ^2^ Department of Linguistics, Faculty of Arts and Philosophy, Ghent University, Ghent, Belgium

**Keywords:** occupational physical activity, physically demanding jobs, need for recovery, social support, job control, accelerometers, sustainable employment

## Abstract

**Objectives:** We investigate whether job control and/or social support at work play a buffering role in the relation between various physical work behaviors and Need for Recovery (NFR) among employees with physically demanding jobs.

**Methods:** Our findings are based on data from 332 workers. The Job Content Questionnaire was used to assess job control, social support and specific physically demanding tasks. General physical work behaviors were measured by two Axivity AX3 accelerometers. The NFR Scale (0–11) was used to assess NFR. We used multiple linear regression models.

**Results:** Sitting at work turned out to be negatively associated with NFR, whereas physically demanding tasks were associated positively with NFR. Our results show a significant buffering role for job control on the correlation between sitting, physically demanding tasks and NFR, but not for social support.

**Conclusion:** Our findings suggest that higher job control might be beneficial to reduce high NFR and eventually may help to reduce early drop-out and sickness absence. Further research is called for to confirm the buffering role of job control and to investigate the underlying mechanisms.

## Introduction

Despite the increasing proportion of sedentary jobs in the working population, physically demanding jobs, such as jobs in the fields of nursing, construction and manufacturing, remain highly prevalent [1]. Previous research has convincingly demonstrated that people with such physically demanding jobs are subject to an increased risk for musculoskeletal problems [2, 3], cardiovascular diseases [4, 5], and all-cause mortality [6]. In order to successfully predict work-related health issues, including the aforementioned three, there are several parameters that can be used, among which “Need for Recovery” (NFR) figures prominently [7, 8]. NFR as a concept has been derived from the Effort-Recuperation Model developed by Meijman and Mulder (1998) [9] and can be defined as the degree to which an employee needs to recuperate both physically and mentally from the effort spent on doing his/her work tasks. It comprises various phenomena, such as temporary feelings of overload, irritability, social withdrawal, lack of energy for new efforts, and reduced performance, especially during the final working hours and in the hours immediately after work [7, 10].

Different studies have shown that workers with a consistently high NFR which is not addressed adequately face an increase in overall health issues [10, 11], including cardiovascular diseases [12]. NFR is furthermore strongly associated with negative phenomena such as fatigue and emotional exhaustion [7], as well as with work absenteeism [10, 13], a reduction in working hours [14], early retirement [15, 16] and occupational disability [16], resulting in high productivity losses for organizations and substantial costs for society [17]. The Effort-Recuperation Model [9] suggests that the reason that periods with high demands should be followed by sufficient recovery in order to prevent adverse health effects is due to the fact that these periods are characterized by high stress levels. Recovery is thus seen as an important element to break a hypothetical causal string where adverse work demands lead to the development of work related stress which in turn leads to several health issues, both mental and physical [11, 13]. NFR is hence a highly relevant factor to take into account in research on sustainable employment and identifying factors associated with an increased NFR is important to develop possible strategies for early prevention of work-related issues [18].

Previous research has demonstrated the prevalence of high levels of NFR in the population of workers with physically demanding jobs [10, 11, 16], e.g., jobs with prolonged standing, frequent stair climbing, continued walking and repetitive movements, in combination with few resting breaks. These employees can be said to deplete their available physical and psychological resources during work [16, 18, 19]. In light of the growing evidence on the harmful effect of physically demanding activity, there is a need for developing preventive measures in order to mitigate these hazards and to propose possible solutions. By relying on the Job-Demand-Control-Support (JDCS) model [20, 21], job control and social support at the workplace can be conceptualized as potential psychosocial moderators to counter the harmful effects of physical work demands [22]. From the model one could derive the hypothesis that states that job control, i.e., “a working individual’s potential control over his/her task and his conduct during the working day,” may prevent job demands from causing negative health outcomes*.* In particular, it has been argued that workplace autonomy can play an important buffering role as it offers employees opportunities to recover, since they can decide for themselves when to take a break. Furthermore, job control also includes facets like skill discretion and decision authority [20]. Skill discretion refers to the possibilities in the workplace for the employees to develop skills so that they can exert control in as many unexpected situations as possible [23, 24]. Workers with such an elevated skill set will be able to deal with their work better due to their increased level of control, perform their tasks without spending excessive amounts of energy and, hence, suffer less from exhaustion. Decision authority refers to the influence of workers over what to do and how to do it. If workers are not forced to do certain tasks in a certain manner, but can decide more for themselves what tasks to do and how to do them, it stands to reason that they can better monitor their own fatigue and adapt their work accordingly. Likewise, a beneficial support system from colleagues and supervisors may also reduce the harmful effect of physical work demands on health [23]. Social support has been described as a social fund that one may draw upon when coping with stressors or job demands, or helpful social interactions that one receives from significant others. The mechanisms behind the beneficial effect of social support could be that the perception of strong social support systems or relationships leads to reduced psychological stress in response to negative events that happen in the workplace [25].

This article therefore aims to examine whether various physical work behaviors are associated with higher experienced NFR in workers with physically demanding jobs and whether psychosocial job resources, i.e., job control and social support, may buffer such a negative relation. Our main hypotheses are that for employees with physically demanding jobs i) increased sitting will be associated with lower NFR, ii) increased standing, MVPA and physically demanding tasks will be associated with higher NFR, and iii) both job control and social support will mitigate high levels of NFR due to a lack of sitting and an excess of standing, MVPA and physically demanding tasks.

Our research is novel on a few fronts. While job control and social support have already been investigated for their direct effect on NFR [19, 26], they have hitherto not been studied as possible moderators of the relationship between physical work behaviors and NFR. Also, we aim at mapping physical work behavior more objectively by applying a combination of accelerometer-based methods and self-reported measures, allowing us to investigate different types of behaviors, including sitting, standing, and moderate-to-vigorous physical activity (MVPA) during work (assessed by two accelerometers), as well as specific physically demanding tasks such as lifting heavy weights, awkward positions, and movements above the head (self-reported).

## Methods

Our study is based on cross-sectional data obtained in the Flemish Employees’ Physical Activity (FEPA) study from February 2017 until June 2018 from 401 employees from 7 different companies in Flanders (Belgium). These companies were all situated in the service and production sector, i.e., a logistics and courier company, a food processing company, a hospital, and four manufacturing companies. All participants met the following inclusion criteria: aged 18–65 years, non-pregnant, Dutch speaking, being employed for at least 50% not having exclusively nightshift work and providing written informed consent prior to participation. Supplementary Figure S1 provides a detailed overview of the recruitment process in our study. The study was approved by the Ethical Committee of Ghent University Hospital (number 2017/0129). Specific details about the protocol have been published previously [27]. For the present analyses, we selected a subsample of 332 employees including only those workers that do not have primarily desk-based jobs and thus excluding workers with sedentary jobs, e.g., administrative workers.

### Measurements

#### Accelerometer-Assessed Physical Work Behaviors

Participants were asked to wear two accelerometers (Axivity AX3), one on the right thigh and one on the back, for up to 2-4 consecutive working days in order to measure physical work behaviors objectively. During the measurement period, participants were asked to fill in a paper-based diary reporting working hours, time of going to and getting out of bed, periods without wearing the monitors, i.e., “non-wear time”, and the daily reference measurement moment, i.e., standing still in a neutral upright position for 15 s.

The accelerometer data were processed using a custom-made MATLAB program (Acti4) for retrieving information with high sensitivity and specificity with regard to physical work behaviors, such as various physical activities and postures, e.g., standing, sitting, walking, stair climbing and running (developed at the National Research Centre for the Working Environment, Copenhagen, Denmark and the Federal Institute for Occupational Safety and Health, Berlin, Germany) [28, 29]. For further analysis, only participants with measurements for both work and leisure time for at least one valid day were included. A valid day was defined as including a minimum of 10 h of data, comprising of least 4 h of work and 4 h of leisure time, or 75% of the average reported work and leisure time. The beginning, duration and end of a work and leisure time period was reported in the diary. Time spent sitting and standing was retrieved directly from the accelerometers. Moderate-to-vigorous physical activity (MVPA) was defined as the time spent running, walking on stairs, and fast walking (>100 steps per minute). All time spent on physical activity during work was expressed as a percentage of the total time at work.

#### Need for Recovery

Need for Recovery (NFR) after work was assessed using a validated 11-item questionnaire [30]. The NFR questionnaire presented 11 dichotomous items (yes/no), related to the recovery after a typical workday, for instance: “At the end of the working day I am really feeling exhausted” and “I find it hard to relax at the end of a working day”. One item, namely “After dinner I usually feel quite fit,” was reversed for scoring. The 11 items were summed up (no = 0; yes = 1) in order to obtain a total score between 0 and 11, whereby a higher score indicates a higher NFR. The scale has previously been evaluated against neuroendocrine activity as well as subjective health complaints [31] and was shown to have good internal consistency [32].

#### Psychosocial Resources and Specific Physically Demanding Tasks

Our detailed assessment of psychosocial work-related factors was based on the Job-Demand-Control-Support (JDCS) model [20]. In particular, the validated Dutch version [33] of the Job Content Questionnaire (JCQ) was used to chart the psychosocial characteristics of the employees involved [34]. The JCQ questionnaire consists of five items (Cronbach *α* = 0.61) for assessing psychological job demands, nine items for job control (*α* = 0.71), divided over six items for skill discretion and three items for decision authority, and eight items to assess social support (*α* = 0.85), consisting of four items to capture supervisor support and four for co-worker support. For our final analysis we grouped the two subtypes of social support, i.e., supervisor and co-worker support, into one general social support parameter, meaning that we did not differentiate between the two subtypes. Separate analyses of supervisor and co-worker support did not reveal any other results than social support taken together. In the results section we therefore only deal with social support as one concept that is construed as the average score over the 8 items of social support. The scale pertaining to specific physically demanding tasks was composed of three items assessing physical exertion, i.e., high physical effort, heavy physical work, and rapid physical activity, and two items assessing isometric loads, i.e., difficult body positions and difficult head or arms positions. All items were scored on a four-point Likert scale ranging from [1], i.e., “completely disagree” to [4], i.e., “completely agree.”

#### Potential Confounders and Baseline Characteristics

In our analyses, we accounted for the potential confounding role of different demographic, work environmental and personal factors, such as age, sex, work schedule [35], psychological job demands, and self-perceived health [18, 35]. Participants who had attended primary school only were classified as having a “low educational level,” those having attended secondary school as a “medium educational level,” and those having completed college or university were considered to have a “high educational level.” Furthermore, height and weight of the participants were measured with a Seca 704 column scale (SECA Medical Measuring Systems and Scales, Birmingham, UK; scales 701/704). The body mass index (BMI) of the participants was calculated as the body weight (kg) divided by the squared height (m).

### Statistical Analyses

Descriptive statistics were presented as means (SD) or as frequencies (%). The distribution of the residuals of the regression followed a normal distribution and the P-P plot followed very closely the diagonal normality line. Based on these tests we can safely assume normality and thus conduct linear analyses. A stepwise linear modelling approach was adopted to investigate the association between various physical behaviors during work, i.e., sitting, standing, MVPA, and self-reported physically demanding tasks, and NFR. Adjustments in the different models were: age and sex (Model 1), Model 1 + work schedule and psychological job demands (Model 2), and Model 2 + self-perceived health (Model 3).

**FIGURE 1:**
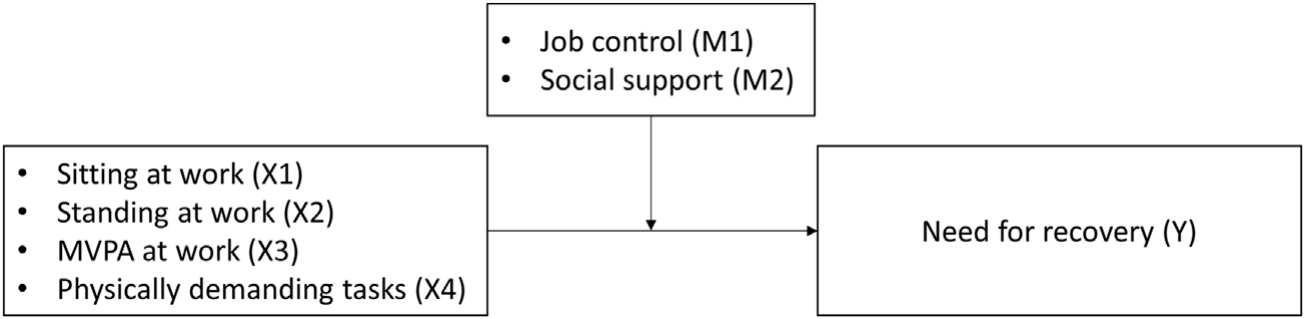
Conceptual diagram showing the moderating effect of job control (M1) and social support (M2) on the relation between physical work behaviors (X1 to X4) and Need for Recovery (Y). (Flemish Employees’ Physical Activity study, Flanders, Belgium. 2017–2018).

The SPSS macro PROCESS (version 3.5) was used to conduct moderation analyses [36] to assess the relation between physical work behaviors and NFR as well as the buffering effect of job control and social support on that relation. Model 1 of the PROCESS package was used, resulting in the estimation of a moderation model with job control and social support as a single moderator. A conceptual diagram for the moderation analyses is shown in Figure 1. Both the predictor and the moderators were mean-centered and were used to form the interaction terms when estimating the moderated path. Mean-centering was done to reduce multicollinearity between the product and its constituent terms [36].

All statistical analyses were conducted using IBM SPSS Statistics Version 26. The level of significance was set at *p* < 0.05.

## Results

Our sample included 190 females and 142 males with an average age of 38.8 (±11.2) years. Most employees worked in the service (49%) or manufacturing (36%) sector, with 64% of all included employees working in shifts. Other information regarding demographics, employment, self-perceived health, psychosocial job factors, and accelerometer-assessed physical behaviors of the participants are provided in Table 1.

**TABLE 1:** Descriptive characteristics of the study population (N = 332). (Flemish Employees’ Physical Activity study, Flanders, Belgium. 2017–2018).

Basic characteristics	Mean (SD) or N (%)
Age (years)	38.8 (11.2)
Sex	
Female	190 (57.2%)
Male	142 (42.8%)
Educational level	
Low (primary school)	59 (17.8%)
Medium (secondary school and/or 1–2 years of specialization)	110 (33.1%)
High (university or university college)	163 (49.1%)
BMI (kg/m^2^)	24.9 (4.2)
Job type (sector)	
Service sector	162 (48.8%)
Skilled worker	24 (7.2%)
Manufacturing sector	119 (35.8%)
Unskilled worker	26 (7.8%)
Work schedule	
Shift	212 (64.2%)
Day job	118 (35.8%)
Workhours per week	36.8 (6.3)
Psychological job demands (1–4 Likert scale)	2.6 (0.5)
Job control (1–4 Likert scale)	2.8 (0.4)
Social support (1–4 Likert scale)	3.0 (0.4)
Physically demanding tasks (1–4 Likert scale)	2.4 (0.6)
Self-perceived health	
Very good	40 (12.2%)
Good	227 (69%)
Fairly good	55 (16.7%)
Poor	7 (2.1%)
Very poor	0 (0%)
NFR (scale from 0 to 11)	3.9 (3.1)
Accelerometer-assessed information	Mean (SD)
Valid accelerometer wear-days	3.0 (0.9)
Mean total work time (min/day)	474 (73.2)
Mean total leisure time (min/day)	466 (100)
Percentage sitting at work	30.5 (21.0)
Percentage standing at work	37.0 (15.9)
Percentage MVPA at work	14.5 (7.3)

BMI, body mass index; SD, standard deviation; N = number of participants; NFR, need for recovery; MVPA, moderate-to-vigorous physical activity.

Table 2 shows the results of the multiple linear regression models of the association between sitting, standing, MVPA, physically demanding tasks and NFR. The unadjusted model demonstrated a significant negative association between sitting at work (B = -0.027; *p* < 0.001) and NFR, whereas standing at work (B = 0.035; *p* = 0.001) and physically demanding tasks (self-reported) (B = 1.852; *p* < 0.001) were both positively associated with NFR. The fully adjusted model revealed similar results for sitting (B = −0.015; *p* = 0.054) and physical demanding tasks (B = 1.171; *p* < 0.001), but the relation between standing at work (B = −0.014; *p* = 0.182) and NFR became non-significant.

**TABLE 2:** Crude and adjusted associations between physical work behaviors (4 separate models) and NFR, results from multiple linear regression analyses in 332 workers. (Flemish Employees’ Physical Activity study, Flanders, Belgium. 2017–2018).

	Unadjusted model	Model 1^a^	Model 2^b^	Model 3^c^
Sitting	**B = -0.027**	**B = -0.023**	**B = -0.017**	**B = -0.015**
	**SE = 0.008**	**SE = 0.008**	**SE = 0.008**	**SE = 0.008**
	***p* < 0.001**	***p* = 0.004**	***p* = 0.032**	***p* = 0.054**
Standing	**B = 0.035**	**B = 0.022**	B = 0.016	B = 0.014
	**SE = 0.011**	**SE = 0.011**	SE = 0.011	SE = 0.011
	***p* = 0.001**	***p* = 0.054**	*p* = 0.135	*p* = 0.182
MVPA	B = 0.025	B = −0.041	B = 0.027	B = 0.027
	SE = 0.024	SE = 0.024	SE = 0.024	SE = 0.024
	*p* = 0.293	*p* = 0.081	*p* = 0.266	*p* = 0.262
Physically demanding tasks	**B = 1.852**	**B = 1.704**	**B = 1.333**	**B = 1.171**
	**SE = 0.247**	**SE = 0.257**	**SE = 0.282**	**SE = 0.284**
	***p* < 0.001**	***p* < 0.001**	***p* < 0.001**	***p* < 0.001**

^a^ Adjusted for age and sex.

^b^ Adjusted for age, sex, work schedule, and job demands.

^c^ Adjusted for age, sex, work schedule, job demands, and self-perceived health.

MVPA, moderate-to-vigorous physical activity; B = regression coefficient; significant associations at *p* < 0.05 are in bold.

Table 3 displays the main effects of physical work behaviors and job control on NFR (i.e., four separate models), as well as their interaction effects. After adjusting for confounders, a significant interaction effect for job control in the relation between sitting (*p* = 0.048), physically demanding tasks (*p* = 0.029) and NFR was found.

**TABLE 3:** Crude and fully adjusted interaction models of the association between physical work behaviors (4 separate models) and job control on NFR, results from PROCESS analyses among 332 workers. (Flemish Employees’ Physical Activity study, Flanders, Belgium. 2017–2018).

	Unadjusted model^a^			Model 3^b^		
	B	SE	*P*	B	SE	*p*
Sitting	**−0.028**	**0.009**	**0.001**	**−0.016**	**0.008**	**0.047**
Job control	0.213	0.367	0.561	0.149	0.346	0.667
Interaction sitting*job control	0.021	0.018	0.256	**0.035**	**0.018**	**0.048**
Standing	**0.034**	**0.011**	**0.002**	0.012	0.011	0.248
Job control	**−**0.123	0.347	0.722	**−**0.091	0.328	0.782
Interaction standing*job control	**−**0.018	0.021	0.401	−0.037	0.021	0.089
MVPA	0.036	0.026	0.172	0.035	0.026	0.172
Job control	**−**0.166	0.357	0.643	**−**0.125	0.336	0.701
Interaction MVPA*job control	0.073	0.49	0.136	0.059	0.045	0.186
Physically demanding tasks	**1.924**	**0.255**	**<0.001**	**1.207**	**0.287**	**<0.001**
Job control	0.161	0.328	0.624	0.109	0.323	0.736
Interaction tasks*job control	−0.837	0.493	0.091	**−1.079**	**0.494**	**0.029**

^a^ Interaction analysis only adjusted for both main effects.

^b^ Interaction analysis adjusted for age, sex, work schedule, job demands, self-perceived health and both main effects.

MVPA, moderate-to-vigorous physical activity; B = regression coefficient; significant associations and interaction effects at *p* < 0.05 are in bold.

**FIGURE 2:**
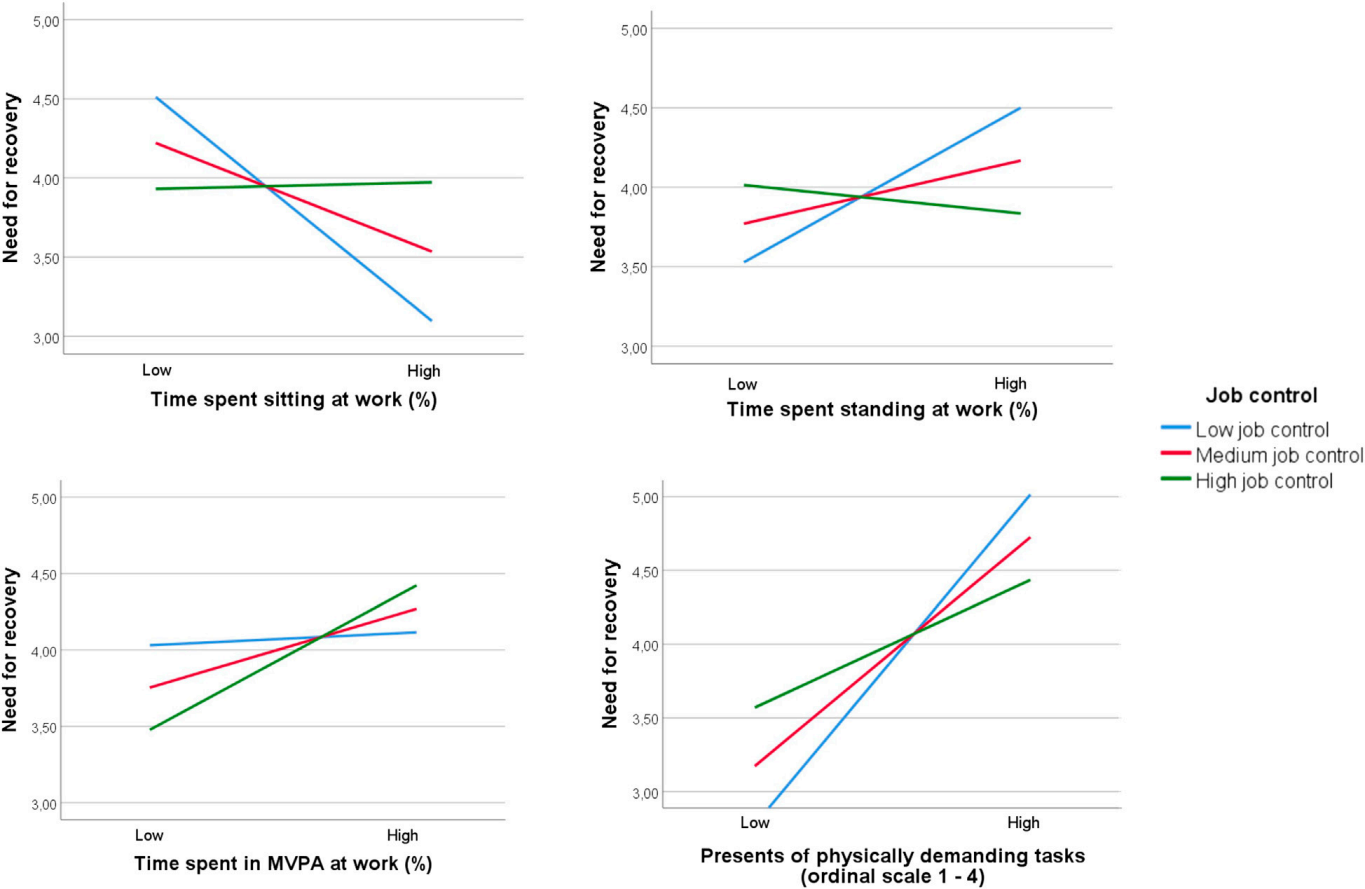
The buffering role of job control on the relationship between physical work behaviors and need for recovery. (Flemish Employees’ Physical Activity study, Flanders, Belgium. 2017–2018).

In order to better understand this significant interaction effect, job control was divided into three levels as displayed in Figure 2. The graphs depict the relation between several physical work behaviors (X1 until X4) and NFR (Y) for different levels of job control (M1). In case job control was at low or medium level, limited sitting was associated with a higher NFR (respectively B = −0.03; *p* = 0.006 and B = −0.02; *p* = 0.05). By contrast, this harmful association was not found when job control was high (B = 0.001; *p* = 0.93). Among workers with low job control, a significant positive association between standing and NFR (B = 0.03; *p* = 0.04) was observed, while in those with medium or high job control the significance of this positive association dropped (respectively B = 0.01; *p* = 0.25 and B = −0.005; *p* = 0.72). The relation between the presence of physically demanding tasks and NFR turned out to be positive for the whole group (low job control: B = 1.74, *p* < 0.001; medium job control: B = 1.21, *p* < 0.001; high job control: B = 0.67, *p* = 0.07). However, the effect size for workers with higher job control was lower. No interaction effects were found for MVPA and job control on NFR and for the interaction effect of social support on the relation between any of the physical behaviors and NFR (Table 4).

**TABLE 4:** Crude and the fully adjusted interaction models between physical work behaviors (4 separate models) and social support on NFR, results from PROCESS analyses in 332 workers. (Flemish Employees’ Physical Activity study, Flanders, Belgium. 2017–2018).

	Unadjusted model^a^			Model 3^b^		
	B	SE	P	B	SE	*p*
Sitting	−**0.026**	**0.008**	**0.001**	−**0.015**	**0.008**	**0.054**
Social support	−0.678	0.391	0.084	0.098	0.391	0.802
Interaction sitting*social support	−0.005	0.02	0.826	0.005	0.019	0.806
Standing	**0.035**	**0.011**	**0.001**	0.015	0.011	0.179
Social support	−0.695	0.385	0.072	0.053	0.385	0.891
Interaction standing*social support	0.021	0.027	0.442	0.009	0.026	0.716
MVPA	0.025	0.024	0.303	0.029	0.024	0.232
Social support	−0.666	0.392	0.090	0.133	0.387	0.731
Interaction MVPA*social support	−0.022	0.052	0.665	−0.044	0.048	0.357
Physical demanding tasks	**1.830**	**0.253**	**<0.001**	**1.199**	**0.286**	**<0.001**
Social support	−0.427	0.364	0.241	0.069	0.375	0.855
Interaction tasks*social support	−0.429	0.523	0.412	−0.545	0.509	0.285

^a^ Interaction analysis only adjusted for both main effects.

^b^ Interaction analysis adjusted for age, sex, work schedule, job demands, self-perceived health and both main effects.

Abbreviations: MVPA, moderate-to-vigorous physical activity; B = regression coefficient; significant associations and interaction effects at *p* < 0.05 are in bold.

## Discussion

Our findings indicate that workers with physically demanding jobs who experience limited time spent sitting and who need to undertake high amounts of physically demanding tasks during work, have difficulties to recover adequately after a working day. At the same time, our data show that this negative correlation seems to be mitigated for those experiencing a higher level of job control. Social support, on the other hand, did not seem to play a buffering role in the negative correlation between sitting, physically demanding tasks and NFR levels.

### Comparison With Previous Studies

Our finding that limited sitting time is associated with increased NFR has also previously been reported in the study of Stevens et al. (2020) [18], which, however, found only a small effect size for this relationship. Our results with regard to the possible positive effect of prolonged sitting on NFR are specific to the group of workers with physically active professions. Increased periods of sitting during the work day for sedentary professions on the other hand has been linked to a series of negative health outcomes, including increased levels of mortality [37], higher risk for cardiovascular disease, cancer and diabetes [38, 39]. For workers with physically active professions increased sitting probably amounts to an increase in the necessary breaks to recover from physically demanding tasks. It remains therefore highly relevant to consider different types of jobs when trying to understand the impact of increased sitting on workers’ health. Both in our study and the one of Stevens et al. (2020) [18] there was no significant association between standing and NFR. A possible explanation for the absence of this association might be that prolonged standing impacts other health related outcomes, such as musculoskeletal pain and increased blood pressure [40], rather than need for recovery which is more related to a general lack of energy. Our fully adjusted model did not show a significant association between objectively measured MVPA and NFR. This is in line with the study of Stevens et al. (2020) [18] where objective measures to capture different physical behaviors pointed to similar results with regard to the impact of MVPA on NFR. The fact that both our studies did not find a significant correlation might be due to the fact that two accelerometers allow us to capture global physical activities, e.g. sitting, standing, lying, walking, running, and stair climbing, but not specific activities such as awkward postures and heavy lifting. Precisely these activities have been shown to have the highest correlations with negative health-related outcomes [1]. Another possible explanation for the lack of significant correlations might be that MVPA during work only takes up a small portion of the work-related physical behaviors and hence has too small an impact.

Our finding that a higher predominance of physically demanding tasks is a risk factor for increased NFR is in line with evidence reported by Bridger et al. 2010 [41] and Gommans et al. (2016) [16]. Both studies showed that increased levels of self-reported physical work demands are indeed correlated with higher levels of NFR. These findings highlight the need to continue research on those specific physically demanding tasks.

Our study is, to our knowledge, the first to indicate the potentially buffering effects of job control on NFR. This finding is in line with results from Sonnentag & Zijlstra (2006) [42], who showed that experiencing task control was directly and negatively associated with NFR. Both findings suggest that taking a break at an appropriate time contributes to reducing NFR and that a reduced workload and enhanced job control may further decrease NFR. Our results that job control played a buffering role in our data is the more interesting because Holtermann et al. 2018 [39] showed that occupational physical activity is typically performed with low job control. They mention limited control over work tasks, speed, schedule, protective clothing, psychosocial stressors and the surrounding environment as factors that may contribute to the detrimental effects of occupational physical activity. Our data show that relevant differences in job control among the group of workers with physically demanding jobs can play a role in lowering the detrimental effects of occupational physical activity.

On the other hand, the fact that social support, does not seem to play a buffering role is somewhat at odds with previous studies, such as Clays et al. (2016) [22], who showed that social support at work can be a considerable effect modifier in the association between physical work demands and cardiovascular disease, and van der Heijden et al. (2010) [43], in which constructive feedback of the supervisor and co-workers were shown to strengthen the ability to deal with job demands and eventually decrease NFR. One possible explanation could be that the meaning and the impact of social support varies across occupations. For instance, it could be that employees with office jobs can benefit more from the social support of a colleague to perform a potentially dull, but not physically demanding, administrative task. Employees with a physically demanding job on the other hand might benefit less from the social/mental support of their colleagues because the work remains physically exhausting, regardless of the social and mental support they receive. Generally speaking, it could also be that personal characteristics play a major role in how social support plays a role. Some employees like social support to a large extent and therefore benefit from it, whereas to other it might seem as unrequested meddling that breaks the individual work flow. If a specific sample of workers is skewed towards the second group of persons, then it is likely that no effect will be found.

### Strengths, Limitations and Recommendations for Future Research

Our study has a number of major strengths. First, the use of objective accelerometer measures to assess physical activity is a major improvement over various previous studies, as it allows to differentiate objectively between several types of physical behaviors during work and thus avoids self-reported bias with regard to physical activity. In order to address the potential shortcoming that accelerometer measures may miss important aspects of some specific physical behaviors, such as lifting a heavy object, we complemented the use of accelerometers with self-reported questionnaires assessing the nature of some of the physically demanding tasks. Second, our sample of 332 workers is relatively large and had a more or less balanced ratio between men and women. Third, this study is to our knowledge the first one to investigate the possible moderating role of job control and social support. Fourth, we took into account multiple confounders to ensure the validity of our models.

Some limitations need to be taken into account as well. First, as we used a cross-sectional design, we cannot determine the causal dynamics of the observed associations. Future studies are therefore needed to investigate factors having a possible causal effect by implementing a longitudinal design. Also, intervention studies that aim to put the potentially causally mitigating role of factors such as job control to the test, are an interesting way forward for future research. A second limitation is that the participating companies and participants were recruited by means of convenience sampling, which might lead to a potential selection bias. A third limitation is that a healthy worker effect may have distorted the findings. Workers who are able to work and to continue working in a challenging work environment are usually the ones that have the necessary physical and mental resilience to continue in that particular context. Fourth, although multiple companies from the industry and healthcare sector were included in this study, it remains uncertain whether these companies constitute a truly representative sample of the overall economic sector. Fifth, there may be limitations associated with the use of self-reported measures of NFR [19], and physiological measures of NFR may be the way forward for future studies [18]. On the other hand, it has been shown that self-reported NFR is strongly correlated with objectively assessed physiological measures of participants, such as cortisol and adrenaline levels [31]. Thus, NFR, as perceived by workers, can be a valid indicator of psychophysiological recovery after work.

### Conclusion

Our study was the first to show the buffering role of job control in the correlation between accelerometer-measured sitting, self-reported physically demanding tasks and NFR. The findings of our study further highlight the importance of enhanced job control as a way to limit potentially harmful effects of occupational physical activity on NFR. Our results confirm the value of prevention at work by focusing on giving workers more freedom with regard to their work schedule, work situation and amount and duration of rest breaks. However, in order to provide evidence-based recommendations, we call for future research that needs to implement longitudinal or intervention designs in the study of accelerometer-measured occupational physical activity and well-measured job control, social support and NFR. These studies may shed light on the possibly causally moderating effect of job control and potentially unravel reveal its underlying mechanism.

## Supplementary Material

Supplementary Figure S1 Flow chart of the recruitment of the study population (Flemish Employees’ Physical Activity study, Flanders, Belgium. 2017–2018).
