# Supplementary figures and images for: Are Psychosocial Resources Buffering the Relation Between Physical Work Behaviors and Need for Recovery?
Source: Int J Public Health. 2022 Dec 16;67:1604787. doi: 10.3389/ijph.2022.1604787 (PMC9800507; doi:10.3389/ijph.2022.1604787)

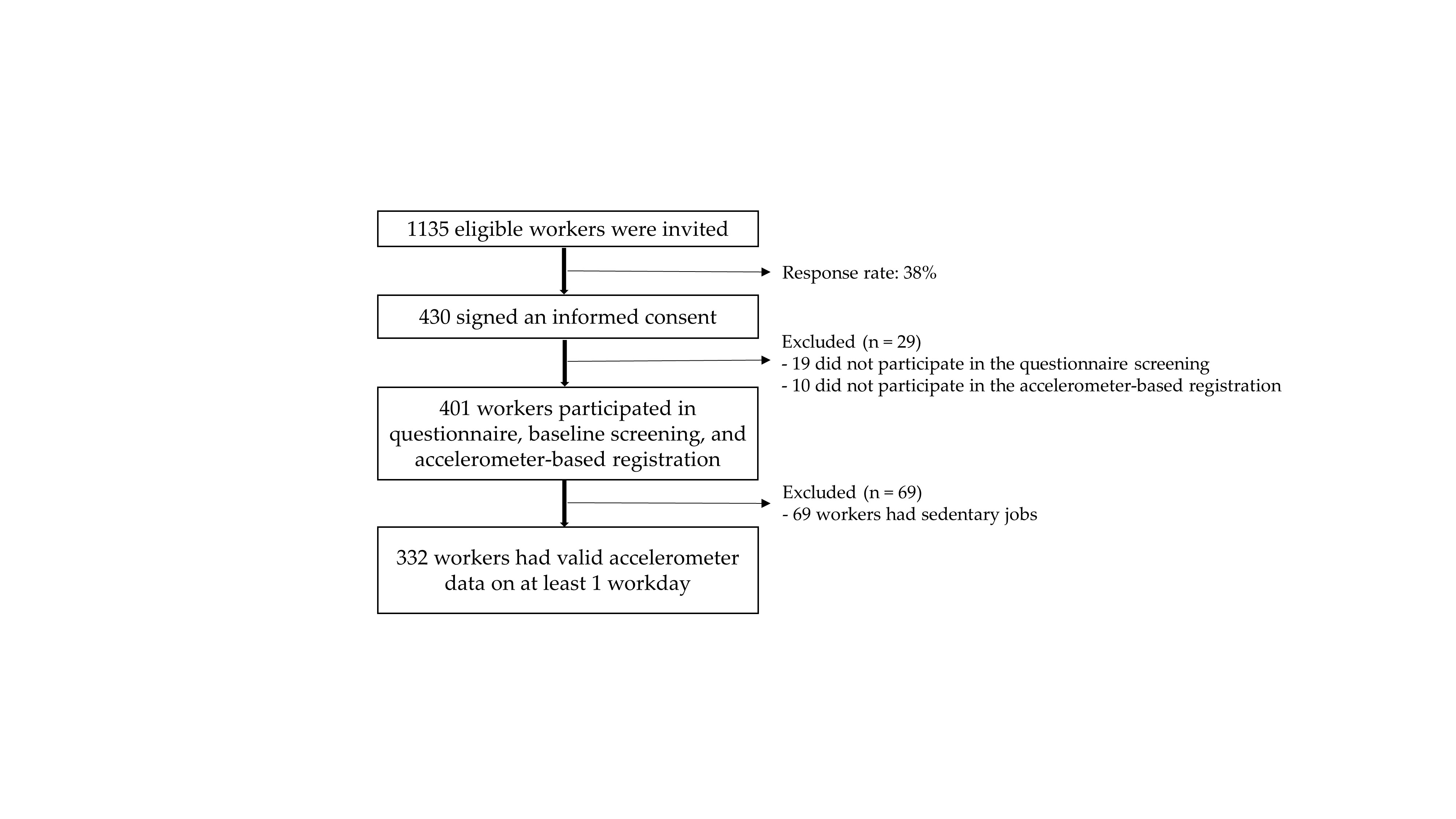

Supplement: Supplementary file 1 [file Image1.JPEG]
